# Fundamental beliefs, as well as levels of depression, anxiety, and stress experienced by Russian students during the second wave of the COVID-19 pandemic

**DOI:** 10.1192/j.eurpsy.2024.1066

**Published:** 2024-08-27

**Authors:** V. I. Rozhdestvenskiy, V. V. Titova, I. A. Gorkovaya, D. O. Ivanov, Y. S. Aleksandrovich

**Affiliations:** Department of Psychosomatics and Psychotherapy, Saint Petersburg State Pediatric Medical University, Saint Petersburg, Russian Federation

## Abstract

**Introduction:**

A pandemic caused by a novel coronavirus is an immensely traumatic event. Researches indicate that such events significantly impact various aspects of individuals, including their physical, emotional, cognitive, behavioural, and social functions, affecting different components of their personality structure. However, the experience of trauma itself is influenced by implicit internal structures known as underlying beliefs. Consequently, emotional responses to traumatic events may be interconnected with these core beliefs.

**Objectives:**

This study aimed to explore fundamental beliefs among Russian university students and analyze their associations with emotional reactions such as depression, anxiety, and stress.

**Methods:**

Data collection took place from January to April 2021 using a custom-designed Google form. The study involved 35 Russian university students. We employed the WAS-37 methodology to investigate fundamental beliefs and the DASS-21 methodology to assess the levels of depression, anxiety, and stress. Both questionnaires were adapted for use in Russia.

**Results:**

We found that the mean values of the “Benevolence of the surrounding world” (M = 35.5±7.3) and “Luck” (M = 31.7±5.1) scales are higher than the normative mean values for the Russian population. In contrast, the mean values of the “Fairness” (M = 21.0±3.7), “Self-image” (M = 26.6±7.0) and “Beliefs about control” (M = 26.6±4.8) scales are generally not different from the normative values. Depression has negative correlations with Self-image (r_s_ = -0.590, p < 0.01) and Beliefs about control (r_s_ = -0.509, p < 0.01). No statistically significant correlations of anxiety and stress with baseline beliefs were obtained.

**Conclusions:**

During the second wave of the pandemic, Russian university students tend to view the world around them as less perilous than the broader population does, and they perceive themselves as more fortunate. Depressive feelings among students are linked to their lower beliefs in the value and importance of their self, as well as their perception that the world around them is not sufficiently controllable.

**Disclosure of Interest:**

None Declared

